# Conservative Management and Early Intensive Rehabilitation Following Haemorrhage of a Thoracic Intramedullary Spinal Cord Cavernoma: A Case Report

**DOI:** 10.7759/cureus.98576

**Published:** 2025-12-06

**Authors:** Dharaneswar Venugopal, Bahar G Yenidunya, Eleonora Bradaschia

**Affiliations:** 1 Rehabilitation Medicine, Orpington Hospital, King's College Hospital NHS Foundation Trust, London, GBR

**Keywords:** cavernous malformation, conservative management, spinal cord cavernoma, spinal cord injury, spinal cord rehabilitation

## Abstract

Spinal cord cavernous malformations (SCCMs) are rare vascular malformations of the spinal cord that may present with acute neurological deterioration in the event of haemorrhage. We report the case of a 21-year-old woman who presented with sudden bilateral lower limb weakness and paraesthesia. She experienced a rapid neurological decline leading to paraplegia and urinary retention. MRI demonstrated a haemorrhagic intramedullary cavernoma at the T12 level with associated cord oedema. Surgical intervention was deferred in the acute phase, given the extent of haemorrhage-related oedema and unclear lesion margins, and conservative management was initiated alongside early intensive neurorehabilitation. The patient subsequently showed significant improvement, progressing from Modified McCormick grade 5 to grade 3 within weeks. By six months, she achieved independent transfers, ambulation with crutches, and functional independence in most activities of daily living, although bladder dysfunction persisted. Follow-up MRI showed a stable lesion with no further haemorrhage. This case illustrates that conservative management with early multidisciplinary neurorehabilitation may facilitate meaningful recovery while neurosurgical planning is underway.

## Introduction

Spinal cord cavernous malformations (SCCMs) are rare vascular lesions of the spinal cord, representing approximately 5-12% of all spinal vascular malformations [1]. They consist of clusters of thin-walled capillaries prone to recurrent micro-haemorrhage and gliosis and may present acutely with neurological deterioration. Clinical manifestations range from back pain and sensory disturbance to motor weakness and bowel and bladder dysfunction, depending on the level of the lesion [2].

While surgical resection is often considered the definitive treatment, the intramedullary location of these lesions makes surgery technically challenging and carries a significant risk of additional neurological morbidity. The role of conservative management remains less clearly defined, largely because the natural history of SCCMs is poorly understood. As highlighted in the literature, most published series are biased toward surgically treated cases, resulting in limited data on non-operative outcomes and making it difficult to develop robust evidence-based guidance [3].

An important consideration in conservatively managed cases is that neurological recovery may arise from a combination of mechanisms. Partial spontaneous improvement may occur as haemorrhage and surrounding oedema resolve, while early neurorehabilitation can further enhance functional recovery. Distinguishing the relative contributions of these factors can be challenging, yet documenting such trajectories is essential for clinicians managing patients in whom surgical intervention poses a high risk.

We present the case of a previously healthy young woman with acute neurological decline secondary to a haemorrhagic spinal cavernoma at T12 who was managed conservatively and demonstrated significant neurological and functional recovery. This case adds to the limited literature supporting a role for non-surgical management and highlights the importance of early multidisciplinary rehabilitation while awaiting further neurosurgical evaluation.

## Case presentation

A 21-year-old female university student with no past medical history presented to the emergency department with acute bilateral lower limb weakness and paraesthesia. She reported waking that morning with pain and altered sensation in both legs and was unable to mobilise. She denied systemic symptoms, recent illness, or trauma. She has not smoked, drunk alcohol, or used recreational drugs. On examination, she was alert and orientated but distressed by her symptoms. Cranial nerves and upper-limb neurological examination were normal. 

In the lower limbs, there was severe bilateral weakness graded 1-2/5 proximally and distally on the MRC muscle-power scale, worse on the right. Reflexes were brisk bilaterally with sustained ankle clonus. Both light touch and pinprick sensations were markedly reduced in the right lower limb compared with the left, with proprioception impaired in the right foot. She subsequently developed urinary retention requiring catheterisation.

**Figure 1:**
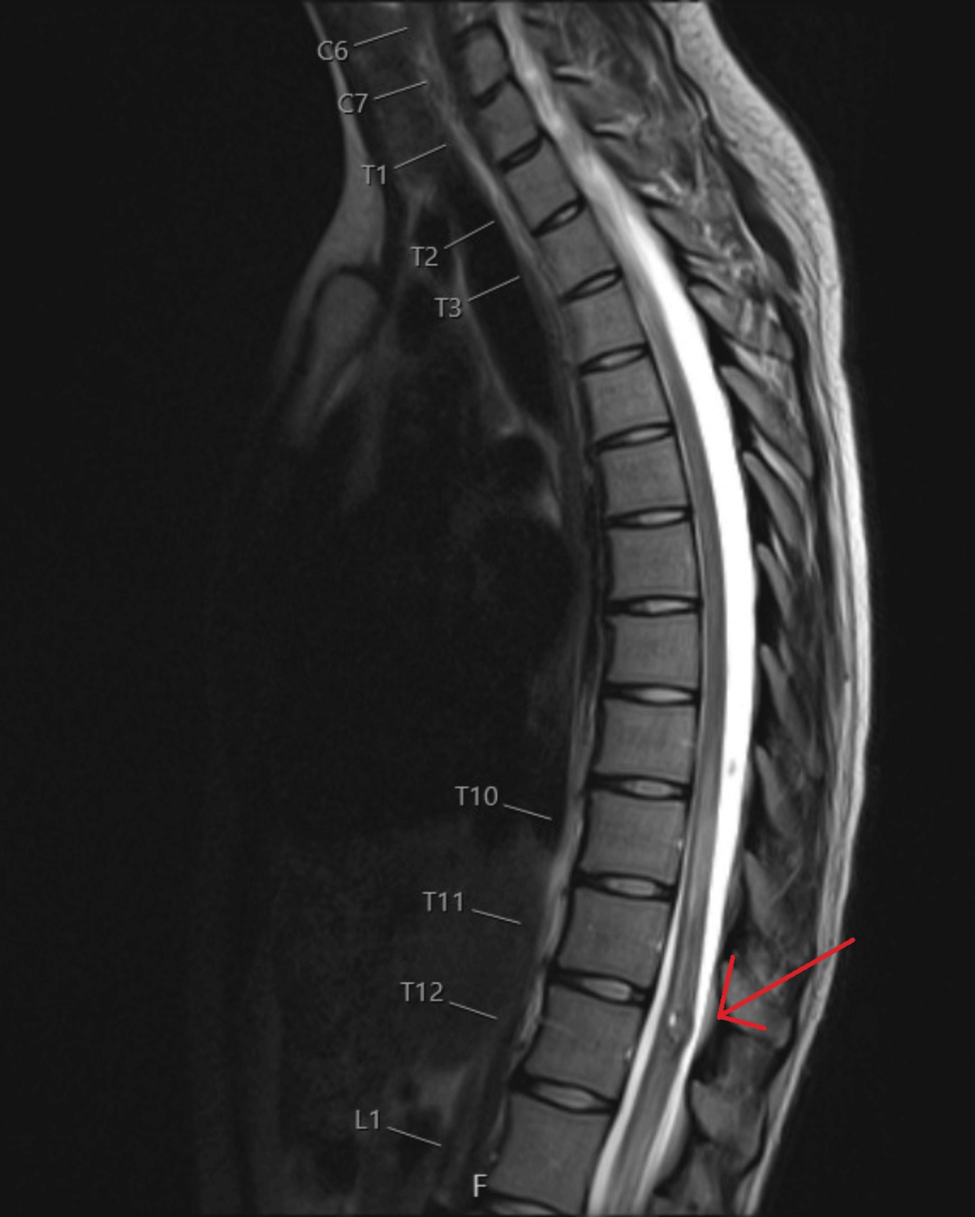
Sagittal T2-weighted MRI of the thoracolumbar spine showing a focal intramedullary lesion at the superior margin of T12 with a surrounding hypointense haemosiderin rim and associated cord oedema extending from T9 to the conus, consistent with a haemorrhagic spinal cavernoma Red arrow mark showing intramedullary iso-intense lesion consistent with a spinal cord cavernoma

**Figure 2:**
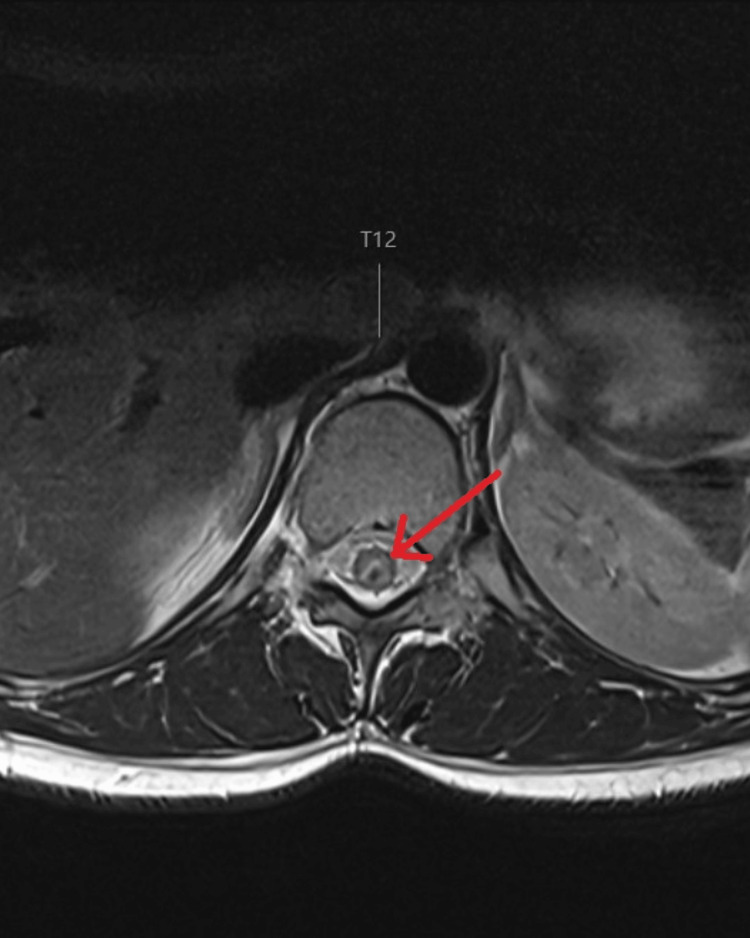
T2-weighted MRI showing axial section of the thoracic spinal cord at T12 level, with dorsally located, isointense lesion with a rim of hyperintense CSF bordering the lesion Red arrow mark showing the intramedullary spinal cord lesion at the level of T12 vertebra

Routine blood tests were unremarkable. MRI of the brain showed no acute pathology. MRI of the whole spine demonstrated a focal intramedullary lesion at the superior margin of the T12 vertebra with a surrounding haemosiderin rim and cord oedema extending from T9 to the conus on the sagittal section (Figure 1), and on the axial section, it showed that the lesion was dorsally located on the spinal cord (Figure 2). The appearances were most consistent with a spinal cavernoma with recent haemorrhage.

She was graded as Modified McCormick Scale grade 5 at presentation. High-dose corticosteroids were commenced, but no immediate benefit was observed, and treatment was discontinued. Following multidisciplinary discussion, surgical intervention was deferred due to the high risk of further cord injury and limited visualisation of the cavernoma in the acute haemorrhagic phase. The plan was to reassess the patient once the haematoma had resolved. In the interim, conservative management was pursued, and she was admitted to a specialist neurorehabilitation unit.

Over the following weeks, gradual neurological recovery was observed. Repeat assessments using the Modified McCormick Scale demonstrated improvement in lower limb function, with partial sensory recovery and an increase in distal power to 4/5 on the Medical Research Council (MRC) scale. Her score improved from grade 5 to grade 3. Although no neurological improvement in bladder function was observed during the initial six-month follow-up, she became independent in intermittent self-catheterisation (ISC) and established reliable bowel management.

With comprehensive multidisciplinary neurorehabilitation, she progressed from requiring hoist-assisted transfers to achieving independent sitting and transfers. She later advanced to standing and mobilising with crutches. At six months of rehabilitation, she is independent in most activities of daily living and is able to mobilise and climb stairs with crutches. Repeat MRI demonstrated a stable lesion with no further haemorrhage. She remains under neurosurgical follow-up and is scheduled for an interval MRI to guide future management.

## Discussion

SCCMs are rare vascular lesions, and much of the current understanding of their natural history is derived from retrospective surgical series. Otten and McCormick emphasised that the published literature is inherently biased towards surgical management, as early reports frequently described resection as both safe and effective. Consequently, conservatively managed patients are markedly under-represented in the literature, limiting insight into the true natural history of these lesions [3].

In a meta-analysis by Badhiwala et al. [2], 631 patients with SCCMs were analysed: 567 (89.9%) underwent surgical resection, while 64 (10.1%) were managed conservatively. The study concluded that, for symptomatic SCCMs, surgery was associated with more favourable neurological outcomes compared with conservative management. However, the authors noted that the relatively small number of conservatively treated patients limited the strength of these conclusions. The natural course of SCCMs therefore remains incompletely characterised, and further reporting of non-operative cases is essential to strengthen the evidence base.

In a small comparative observational study, Kharkar et al. [4] followed 14 patients with intramedullary SCCMs, 10 managed conservatively and four treated surgically, over a mean follow-up of 42 months. Among the conservatively managed group, neurological status remained largely stable, with no significant progression of deficits despite many patients having pre-existing myelopathy and motor weakness at baseline. The authors concluded that conservative management may be appropriate for patients with mild or stable deficits, reserving surgery for those with progressive neurological deterioration.

Additional case reports have described favourable outcomes without surgery. Mikula et al. [5] reported a 28-year-old patient with a large cervical intramedullary cavernoma (C2-C7) who demonstrated spontaneous regression on follow-up imaging, a rare but striking outcome, suggesting that haemorrhagic lesions may occasionally undergo self-resolution.

Similarly, Gupta et al. [6] described a 45-year-old man with a cervical SCCM who declined surgery and was managed conservatively with physical therapy, analgesia, and regular radiological surveillance. Caution was taken to monitor the patients closely and to repeat an MRI at the first sign of worsening neurology. However, this patient remained without any progressive symptoms. The authors emphasised the importance of close follow-up with MRI every 6-12 months and urgent imaging if new or worsening neurological symptoms occur.

A large systematic review by Asimakidou et al. [7] analysed 1,091 patients with intramedullary SCCMs, comparing surgical and conservative management. Of these, 1,005 underwent surgery, and 86 were managed conservatively. Among conservatively treated patients, 21.7% improved, 69.6% remained stable, and 8.7% deteriorated. In the surgical cohort, 36.9% improved, 55.8% remained stable, and 7.3% deteriorated. Although surgical outcomes appeared more favourable overall, the comparable rates of neurological deterioration between the two groups suggest that conservative management may be a reasonable option in selected cases. It is important to note here as well the substantial discrepancy in sample sizes between the surgical and conservative cohorts, which may limit the validity of direct statistical comparison and interpretation of the results.

What differentiates this case from previously published reports is the severity of presentation and the context in which conservative management was used. Most non-operative SCCM cases describe mild or stable symptoms, whereas our patient presented with profound impairment (Modified McCormick grade 5) following acute haemorrhage. Despite this severity, she improved to grade 3 within weeks and achieved independent mobility by six months. Furthermore, neurorehabilitation in this case was not used as a substitute for surgery, but as an interim strategy while awaiting radiological stabilisation and more accurate assessment of operability. This trajectory demonstrates that meaningful recovery may occur during conservative management when surgery is deferred due to a plethora of reasons, in this case, while awaiting hematoma resolution.

This case, therefore, expands current evidence by showing that early multidisciplinary neurorehabilitation may facilitate neurological improvement during an interim observation period, even in patients with severe initial deficits. Continued documentation of conservatively managed SCCMs is needed to clarify recovery trajectories and support clinical decision-making in cases where immediate surgery is not undertaken.

## Conclusions

This case demonstrates that meaningful neurological and functional improvement may occur during a period of conservative management in a patient with haemorrhagic SCCM, particularly when combined with early and intensive multidisciplinary neurorehabilitation. In this instance, conservative treatment served as an interim approach while awaiting radiological stabilisation and further neurosurgical assessment, rather than as a definitive alternative to surgery. Although a single case cannot establish the efficacy of non-operative management, it contributes a valuable clinical observation to the limited literature and highlights neurorehabilitation as a key supportive component during non-surgical monitoring. Further accumulation of similar cases and prospective data will be necessary to clarify which patients may be most suitable for initial conservative management, how neurorehabilitation influences recovery trajectory, and under what clinical conditions delaying surgery may be appropriate.
